# Prevalence and Factors Associated with Intestinal Parasitic Infection among Children in an Urban Slum of Karachi

**DOI:** 10.1371/journal.pone.0003680

**Published:** 2008-11-10

**Authors:** Vikram Mehraj, Juanita Hatcher, Saeed Akhtar, Ghazala Rafique, Mohammad Asim Beg

**Affiliations:** 1 Department of Pathology & Microbiology, Aga Khan University, Karachi, Pakistan; 2 Department of Community Health Sciences, Aga Khan University, Karachi, Pakistan; 3 Unité Mixte de Recherche 6236, Centre National de la Recherche Scientifique, Faculté de Médecine, Université de la Méditerranée, Marseille, France; 4 Department of Community Medicine and Behavioral Sciences, Faculty of Medicine, Kuwait University, Safat, Kuwait; London School of Hygiene & Tropical Medicine, United Kingdom

## Abstract

**Background:**

Intestinal parasitic infections are endemic worldwide and have been described as constituting the greatest single worldwide cause of illness and disease. Poverty, illiteracy, poor hygiene, lack of access to potable water and hot and humid tropical climate are the factors associated with intestinal parasitic infections. The study aimed to estimate prevalence and identify factors associated with intestinal parasitic infections among 1 to 5 years old children residing in an urban slum of Karachi Pakistan.

**Methods and Principal Findings:**

A cross sectional survey was conducted from February to June 2006 in Ghosia Colony Gulshan Town Karachi, Pakistan. A simple random sample of 350 children aged 1–5 years was collected. The study used structured pre-tested questionnaire, anthropometric tools and stool tests to obtain epidemiological and disease data. Data were analyzed using appropriate descriptive, univariate and multivariable logistic regression methods. The mean age of participants was 2.8 years and 53% were male. The proportions of wasted, stunted and underweight children were 10.4%, 58.9% and 32.7% respectively. The prevalence of Intestinal parasitic infections was estimated to be 52.8% (95% CI: 46.1; 59.4). *Giardia lamblia* was the most common parasite followed by *Ascaris lumbricoides*, *Blastocystis hominis and Hymenolepis nana*. About 43% children were infected with single parasite and 10% with multiple parasites. Age {Adjusted Odds Ratio (aOR) = 1.5; 95% CI: 1.1; 1.9}, living in rented households (aOR = 2.0; 95% CI: 1.0; 3.9) and history of excessive crying (aOR = 1.9; 95% CI: 1.0; 3.4) were significantly associated with intestinal parasitic infections.

**Conclusions:**

Intestinal parasites are highly prevalent in this setting and poverty was implicated as an important risk factor for infection. Effective poverty reduction programmes and promotion of deworming could reduce intestinal parasite carriage. There is a need for mass scale campaigns to create awareness about health and hygiene.

## Introduction

Intestinal parasitic infections (IPIs) are globally endemic and have been described as constituting the greatest single worldwide cause of illness and disease [1]–[3]. IPIs are linked to lack of sanitation, lack of access to safe water and improper hygiene; therefore they occur wherever there is poverty. IPIs deprive the poorest of the poor of health, contributing to economic instability and social marginalization. The poor people of under developed nations experience a cycle where under nutrition and repeated infections lead to excess morbidity that can continue from generation to generation. People of all ages are affected by this cycle of prevalent parasitic infections; however, children are the worst affected [1], [2].

*Ascaris lumbricoides*, *Trichuris trichiura* and hookworms, collectively referred to as soil-transmitted helminths (STHs), are the most common intestinal parasites. [4]. *Ascaris lumbricoides* is the largest and the most common helminth parasitizing the human intestine and currently infects about 1 billion people worldwide [5]. *Hymenolepis nana* is the most common parasitic cestode prevalent globally [6]. *Giardia duodenalis/Giardia intestinalis*, previously known as *Giardia lamblia*, causing giardiasis, is the most prevalent protozoan parasite worldwide with about 200 million people being currently infected [6], [7]. Another common intestinal protozoan is *Blastocystis hominis* whose parasitic status is under debate [5].

About one third of the world, more than two billion people, are infected with intestinal parasites [8], [9]. Approximately 300 million people are severely ill with these worms and of those, at least 50% are school-age children [8]. IPIs rarely cause death but because of the size of the problem, the global number of related deaths is substantial [10]. About 39 million disability adjusted life years (DALYs) are attributed to IPIs and these infectious thus represent a substantial economic burden [11].

This research was aimed at estimating the prevalence of intestinal parasites and its covariates among children from 1–5 years of age residing in an urban slum of Karachi

## Methods

### Study Design and Setting

This cross-sectional survey was carried out from February to June 2006 in Ghosia Colony, a squatter settlement in the central area of Karachi which is the largest city of Pakistan consisting of 10% of the total population. As per the last census conducted in 1998, the population of Karachi was about 9.8 million [12]. With an estimated current population of around 15 million, it is one of the most populous cities of the world with a high rate of population growth [13] mainly due to rapid urbanization.

### Study Population, Sample Size and Sampling Strategy

The study population consisted of all children from 1 to 5 years of age residing in the households of Ghosia Colony [14]. The sample size was calculated for the primary objective taking the prevalence to be estimated of 50% that gives the maximum sample size, with 95% level of confidence and 5% bound on the error of estimation. The minimum sample size required was 385 children. Simple Random Sampling method was used to select the households. A list of all the households (the sampling frame) was prepared in a preliminary census type survey. Among all those households where more than one eligible child was present, one was randomly selected through simple lottery method. A total of 1425 children were present in the sampled households and of which about 27.1% were randomly selected.

### Date Collection

The outcome variable was stool parasite status of the selected child, whether positive or negative for any intestinal parasite, which was determined from a stool sample. Data on the independent variables, socio-demographic characteristics, mother and child behavioral characteristics and past medical history were collected by trained research officers on a pre-tested and structured questionnaire addressed to the mother. Height and Weight of the selected children were also measured using standard calibrated instruments.

### Stool Sample Collection and Laboratory Testing

For each selected child in the study, a standard stool ova and parasite test with one concentration technique was done for the assessment of outcome [5]. The test was conducted at the Juma Research Laboratory, Aga Khan University by the principal investigator and an experienced laboratory technologist. At the time of interview, the mothers were given a dry, clean, leak proof container labeled with the name and identification number for the collection of stool sample of the child the next day. The mothers were also guided on how to collect the sample. Either the stool was to be collected directly as the child defecates or a small piece of the feces was to be put into the sample bottle after the child defecates, through the help of a wooden spoon already provided with the collecting container. Field staff were trained in proper hygienic and bio-safety measures. In the laboratory, slides were then prepared directly for wet mount in saline as well as in iodine and then were microscopically examined initially under low power (10× = 100 times magnification) bright field then under high power (40× = 400 times magnification) bright field. Finally the sample was concentrated applying the formalin-ethyl acetate technique [5] and again the iodine stained slides were prepared and examined microscopically. The stool test positive children were also provided with anti-parasitic treatment. Albendazole and/or metronidazole [5] were given as appropriate under consultation of a physician.

### Ethical Considerations

As the participant children to be included were 1 to 5 years of age therefore the informed consent was sought from the mothers. The ethical approval of the study was sought and obtained from the Aga Khan University Ethical Review Committee (ERC) and University of Alabama Institutional Review Board.

### Data Management and Statistical Analysis

During data collection completed questionnaires were checked regularly to rectify any discrepancy, logical errors or missing values. The data entry was carried out using Epiinfo 6.04d software [15] and then the data was exported to Statistical Package for the Social Sciences (SPSS) [16] for statistical analysis.

Variables were categorized in a biologically meaningful way where applicable. Age, height and weight were used to calculate the anthropometric indices; i.e. stunting (height for age), wasting (weight for height) and underweight (weight for age) through software WHO Anthro [17].

To describe data, mean & standard deviation for continuous variables and proportion for categorical variables were computed. Crude associations of the binary outcome variable with each independent variable were assessed by student's t-test or chi square test as appropriate. The level of statistical significance was set as p≤0.05 and for each statistically significant factor, an odds ratio and 95% confidence interval (CI) was computed by the univariate logistic regression analysis.

A multiple logistic regression analysis was run to study the independent association of variables with IPIs. The level of significance of p≤0.05 was set for multivariable analysis. The final model was tested for goodness-of-fit by applying the Hosmer-Lemeshow Test. The final model was interpreted by using adjusted ORs and 95% CIs.

## Results

The results represent information collected from 350 interviews and 218 stool samples with a response rate of 62.3%.

### Prevalence of Intestinal Parasitic Infections (IPIs)

The overall prevalence (95% CI) of the IPIs was estimated at 52.8% (46.1%; 59.4%), i.e. 115 positive out of 218. About 43% of samples contained a single parasite and 10% contained multiple parasites. *Giardia lamblia*, being the most common IP, was present in 63 samples (28.9%) followed by *Ascaris lumbricoides* present in 36 samples (16.5%), *Blastocystis hominis* in 22 samples (10.1%), *Hymenolepis nana* in 2 samples (0.9%), *Endolimax nana* in 4 samples (1.8%), *Entamoeba coli* in 5 samples (2.3%) and *Iodoamoeba butschlii* in 7 samples (3.2%) were identified from the stool samples. (Table 1)

**Table 1:** Prevalence of intestinal parasitic infections among children, 1 to 5 years of age, residing in Ghosia Colony, Karachi, Pakistan (n = 218).

Characteristic	n	%	95% CI	
			Lower	Upper
**Intestinal Parasitic Infection**	115	52.8	46.1	59.4
Multiple¥	21	9.6	5.7	13.5
Single†	94	43.1	36.5	49.7
Nil	103	47.2	40.6	53.9
***Giardia lamblia***	63	28.9	22.9	34.9
Mono parasitism¶	52	23.9	18.2	29.5
Poly parasitism*	11	5.0	2.1	8.0
***Ascaris lumbricoides***	36	16.5	11.6	21.4
Mono parasitism¶	25	11.5	7.2	15.7
Poly parasitism*	11	5.0	2.1	8.0
***Blastocystis hominis***	22	10.1	6.1	14.1
Mono parasitism¶	15	6.9	3.5	10.2
Poly parasitism*	7	3.2	0.9	5.6
***Hymenolepis nana***	2	0.9	-	-
Mono parasitism¶	0	0	0.0	0.0
Poly parasitism*	2	0.9	-	-

¥ If more than one intestinal parasite is identified in a sample, it is a multiple parasitic infection.

† If only one intestinal parasite is identified in a sample, it is a single parasitic infection.

¶ Infection with the labeled parasite only.

* Infection with multiple parasites including the labeled one.

In 13.9% of the Ascaris positive samples (5 out of 36) co-infection with Giardia was also observed and this association was statistically significant (p = 0.030). (Data not shown)

### Descriptive Characteristics

Fifty three percent of the children were male. The mean (±SD) age of the children was 2.8±1.1 years. The total number of children born to the interviewed mothers ranged from 1 to 12 with an average of 3.7±1.9 (Mean±SD). Thirty seven percent of mothers and 55% of fathers were educated above primary (5^th^ class). The mean (±SD) monthly family income (in Pakistan Rs.) was 6841±3926 with a median (±IQR) of 6000 (4000). About a quarter of the households were rented and the rest were owned. The proportions of children categorized as wasted, stunted and underweight were 10.4%, 58.9% and 32.7% respectively. Geophagia (Soil eating habit), in participant children was reported by 24.8% of the mothers. A few factors related with the environment such as type of latrine, type of household construction, source of drinking water and type of sewage line did not show variation therefore were not analyzed further.

### Crude Associations of independent variables with IPI

**Figure 1:**
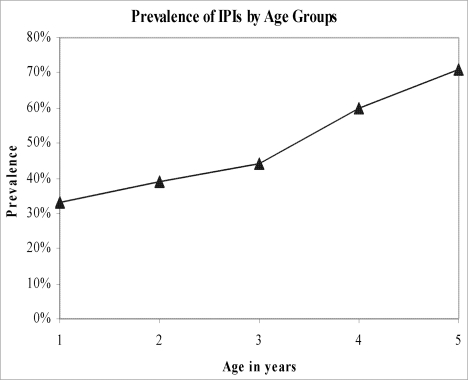
Prevalence of IPIs by age groups.

With each year increase in age the odds of IPIs increased by 1.7 times (95% CI: 1.3; 2.1). Children living in rented households were more likely to be infected with intestinal parasites (IPs) as compared to the children living in their own households (OR = 1.5; 95% CI: 1.2; 1.9). The children, who cared for defecating themselves, were more likely (OR = 2.5; 95% CI: 1.4; 13.8) to be infected with IPIs compared to children being cared for defecating by their mothers. Positive history of diarrhea in the past month was inversely associated with IPIs (OR = 0.5; 95% CI: 0.3; 0.9). History of excessive crying was significantly more common in infected children (OR = 1.7; 95% CI: 1.0; 3.0) (Table 2 & Figure 1)

**Table 2:** Univariate analysis of factors associated with intestinal parasitic infection among children, 1 to 5 years of age, residing in Ghosia Colony, Karachi, Pakistan who provided a stool sample for this study.

Characteristics	Positive = 115	Negative = 103	OR	95% CI	p
	n (%)	n (%)			
**Gender**					0.255
Male	57 (49.6)	59 (57.3)	-	-	
Female	58 (50.4)	44 (42.7)	1.4	0.8; 2.3	
**Age in years**					<0.001*
(Mean±SD)	3.1 (1.1)	2.5 (1.1)	1.7	1.3; 2.1	
**Education of mother**					0.059¶
Above primary	36 (31.3)	45 (55.6)	-	-	
Primary & below	79 (68.7)	58 (42.3)	1.7	0.9; 2.9	
**Mother's working status**					0.862
Working	20 (17.4)	17 (16.5)	-	-	
Non-working	95 (82.6)	86 (83.5)	0.9	0.5; 1.9	
**Monthly family income**					0.096¶
(Mean±SD)	6405 (2955)$	7336 (4764)∼	1.0	1.0; 1.0	
**Rented household**					0.004*
No	74 (64.3)	84 (81.6)	-	-	
Yes	41 (35.7)	19 (18.4)	1.5	1.2; 1.9	
**Wasting** (Weight for Height)†					0.136¶
Normal	100 (92.6)	81 (86.2)	-	-	
Wasted	8 (7.4)	13 (13.8)	0.5	0.2; 1.3	
**Stunting** (Height for Age)†					0.642
Normal	46 (42.6)	37 (39.4)	-	-	
Stunted	62 (57.4)	57 (60.6)	0.9	0.5; 1.5	
**Underweight** (Weight for Age)†					0.492
Normal	75 (69.4)	61 (64.9)	-	-	
Underweight	33 (30.6)	33 (35.1)	0.8	0.5; 1.5	
**Latrine care**					0.008*
Mother	91 (79.1)	96 (93.2)	-	-	
Others	7 (6.1)	3 (2.9)	2.5	0.6; 9.8	
Self	17 (14.8)	4 (3.9)	4.5	1.5; 13.8	
**Geophagia**					0.154¶
No	82 (71.3)	82 (79.6)	-	-	
Yes	33 (28.7)	21 (20.4)	1.2	0.9; 1.6	
**History of diarrhea in past month**					0.024*
No	60 (52.2)	38 (36.9)	-	-	
Yes	55 (47.8)	65 (63.1)	0.5	0.3; 0.9	
**History of abdominal pain**					0.193¶
No	58 (50.4)	61 (59.2)	-	-	
Yes	57 (49.6)	42 (40.8)	1.4	0.8; 2.4	
**History of excessive crying**					0.048*
No	62 (53.9)	69 (67.0)	-	-	
Yes	53 (46.1)	34 (33.0)	1.7	1.0; 3.0	

$ n = 103.

∼ n = 91.

† n = 202.

* p≤0.05.

¶ p≤0.25.

### Multivariable Analysis

The final model indicated that age of the child, positive history of excessive crying of the child and rented household by the family were significantly associated with the IPIs. With each year increase in age the adjusted odds ratio of IPIs increased by 1.5 times (95% CI: 1.1; 1.9). The children of families living in rented households were more likely to be infected with IPIs as compared to children of families living in their own households (AOR = 2.0 times; 95% CI: 1.0; 3.9). Children with a history of excessive crying were also more likely to be infected with IPIs (AOR = 1.9 times; 95% CI: 1.0; 3.4). Two other variables, latrine care and history of diarrhea in past month, were not significantly associated with the IPIs but kept in the final model because of their confounding effect. The Hosmer-Lemeshow goodness-of-fit test for the final model showed reasonable fit (χ^2^ = 6.132, p = 0.632).

## Discussion

We estimated the prevalence of intestinal parasitic infections (IPIs) and factors associated with IPIs among children 1–5 years of age from an urban slum of Karachi. This study also showed that age of the child, rented household and history of excessive crying were significantly associated with intestinal parasitic infection (IPIs).

The prevalence of IPIs was estimated to be 52.8% and such high prevalence has been consistently reported by a number of studies conducted in similar populations [18]–[24].

In this study the intestinal parasites namely *Giardia lamblia*, *Ascaris lumbricoides*, *Blastocystis hominis*, *Hymenolepis nana*, *Endolimax nana*, *Entamoeba coli* and *Iodoamoeba butschlii* were identified from the stool samples. Coinfection with Trichuris was not seen in our urban slum population although this is commonly seen. The transmission of Ascaris/Trichuris infections are generally more in rural areas however in urban slums, the transmission is probably related to poor sanitary conditions or contaminated water supplies. Perhaps Trichuris cannot successfully complete its life cycle in the absence of a more soil rich rural environment and may well be less adapted to conditions in an urban slum for successful transmission. A statistically significant association between Ascaris and Giardia, found in the current study, is due to the common environmental factors which may affect their transmission.

Our diagnostic sensitivity may have improved if we had taken three consecutive stool samples but this was not done.

No hookworm was identified in our study which is consistent with results obtained in studies [18], [25] conducted in urban localities but not with others [20], [26] conducted in rural areas. This shows that the parasitic profile of urban slums is not quite similar to that in rural areas and further exploration is required to make conclusions.

We could not determine any association between the anthropometric indices and IPIs and there is a discrepancy in the available literature on this question as some studies reported a positive association [4], [11], [27] whereas others reported no association [28]–[30]. In our study this may be explained by the small sample size and other limitations of the study or owing to a real absence of measurable difference. However, further prospective studies should be conducted before making any conclusion.

Age is an important risk factor for IPIs [31] and the pre-school and school going children have been reported to be at highest risk for IPIs [4]. In our study, we have identified an increasing dose-response association between age and IPIs within the age group of 12 to 60 month. This could be due to the fact that as the child grows older the exposure to many of the risk factors for IPIs increases. The linear association of age within this range needs further exploration through prospective studies.

Lower socioeconomic status (SES) is also a risk factor for IPIs [32]. We have taken rented house as a proxy measure of SES which is also positively associated with IPIs. The effect of SES on risk of infectious diseases in general, and parasitic infections in particular, is complex in nature and could be attributed to several other factors such as lack of access to clean water, poor hygienic environment, lack of access to education due to financial constraints and overcrowded conditions [33], [34].

We found history of excessive crying to be positively associated with IPIs. Like many other diseases diarrhea causes irritation and the suffering children are expected to cry excessively [35]–[37]. Further exploration is required to link excessive crying to hunger, either due to poverty (and therefore not an independent risk factor) or due to intestinal worm burden reducing nutrition.

### Strengths and Limitations

As per our knowledge, this is the first study in Karachi Pakistan focusing on children of urban slum areas. The stool sample testing by routine ova and parasite method and a concentration technique increased the validity of the estimates.

Our study has certain limitations that need to be considered while interpreting results.

The response rate was lower than expected (62%) limiting the number of samples obtained but, since the characteristics of respondents and non-respondents were comparable we would expect the results to be representative of the community.

We conducted single stool examination for detection of intestinal parasites, which could have underestimated the prevalence, as optimal laboratory diagnosis of IPIs requires the examination of at least three stool specimens collected over several days. More recent studies have suggested that one or two stool samples will detect up to 90% of the protozoa present [38], [39].

It was planned to conduct stool sample testing within two hours of collection, however, due to logistic constraints, it was delayed at times from three to six hours as a result of which we could not detect the invasive form of protozoans, i.e. the trophozoites.

We can not drawn conclusions on causality of associations of different factors with IPIs as this is a limitation in cross sectional study design.

### Conclusion

Intestinal parasitic infections are highly prevalent in urban slum areas in this setting. Poverty is an important factor associated with IPIs and the government should enhance the activity of poverty reduction programs. Age is an important predicator of IPIs in 1 to 5 year old children with excessive history of crying. There is a need to promote mass scale deworming and health promotion campaigns to create awareness about health and hygiene.
